# Lifetime Victimization and Physical Health Outcomes among Lesbian and Heterosexual Women

**DOI:** 10.1371/journal.pone.0101939

**Published:** 2014-07-28

**Authors:** Judith P. Andersen, Tonda L. Hughes, Christopher Zou, Sharon C. Wilsnack

**Affiliations:** 1 University of Toronto Mississauga, Department of Psychology, Mississauga, Ontario, Canada; 2 University of Illinois at Chicago, Department of Health Systems Sciences, College of Nursing, Chicago, Illinois, United States of America; 3 University of North Dakota, Department of Clinical Neuroscience, School of Medicine & Health Sciences, Grand Forks, North Dakota, United States of America; University of Utah, United States of America

## Abstract

**Background:**

Lifetime victimization experiences, including child sexual abuse (CSA), child physical abuse (CPA), adult sexual assault (ASA), and adult physical assault (APA), are associated with health problems.

**Purpose:**

To examine relationships between cumulative victimization and physical health among heterosexual and lesbian women and determine whether these relationships differ by sexual identity.

**Methods:**

Large samples of heterosexual (n = 482) and lesbian women (n = 394) were interviewed. Questions included lifetime victimization experiences and physical health problems.

**Results:**

Compared to women who reported no childhood victimization, those who reported experiencing both CSA and CPA were 44% more likely to report health problems and women who experienced all four types of victimization (CSA, CPA, APA, ASA) were nearly 240% as likely to report physical health problems. Interaction analyses revealed the association between victimization and physical health did not differ by sexual identity.

**Conclusions:**

Although lesbians were more likely to report all types of victimization, results suggest that victimization conferred increased physical health risks regardless of sexual identity.

## Introduction

According to the National Center for Injury Prevention and Control, 46% of US women have experienced rape or some other type of forced sexual contact in their lifetime [1]. Using population-based data, the US Department of Justice estimates that over 40% of US women have experienced physical abuse from a caregiver while growing up and that 22% of women experience intimate partner violence at some point in their lifetime [2]. Recent meta-analytic reviews provide evidence that childhood abuse is associated with a wide range of physical health problems including, most commonly, gastrointestinal and gynecological conditions, cardiovascular disease, metabolic disorders, cancer, and pain conditions [3], [4], [5]. Recent research suggests that sexual minority individuals (i.e., lesbian, gay, bisexual) experience higher prevalence of sexual and physical abuse in childhood, and sexual and physical assault in adulthood then their heterosexual peers [6], [7], [8]. In this study, we extend the literature on the health impact of victimization by examining the association between lifetime victimization and physical health problems in both heterosexual and lesbian women.

### Lifetime Victimization and Physical Health

Research shows that both childhood and adulthood victimization can confer health risks. Abuse has been shown to confer profound negative neurobiological consequences during childhood development [9]. Mechanisms by which child physical and sexual abuse confer negative health risks include disruptions in stress response physiology, stress hormones, and immune function, which may have serious implications for a child's ability to respond adaptively to future stress [10], [11]. Population-based studies provide ample evidence of the association between child abuse and health problems in adulthood. In a large, population-based sample, Springer and colleagues found a 21% increase in medical diagnoses and a 22% increase in physical symptoms among adults reporting childhood physical abuse compared to non-abused peers [12]. Multiple forms of abuse and assault appear to compound the risk of physical health problems across the lifespan [3], [13]. Felitti and colleagues found a twofold increase in odds of ischemic heart disease, cancer, and stroke among individuals reporting four or more adverse childhood experiences [14].

Victimization during adulthood, even single-incident victimization events such as rape or assault, has been shown to negatively impact physical health across the lifespan [15]. Individuals who experience both severe childhood and adult adversity may be even more at risk for poor physical outcomes. Fagundes and colleagues [3] examined the interaction between childhood adversity (parental abuse/assault) and severe adverse events in adulthood among 91 patients with a basal cell carcinoma (BCC). The authors found that the combination of experiencing both child and adulthood adversity predicted a lowered immune response to the BCC tumor. This research provides evidence for mechanisms by which cumulative adversity may impact lifetime health. Further, since victimization experienced in childhood may shape an individual's responses to future stress, it is important to examine the association of childhood and adulthood victimization on physical health independently from one another, as well as their cumulative impact [16]. Given past research findings, we expect to find an additive effect of childhood and adulthood victimization on physical health problems among heterosexual and lesbian women.

### Lifetime Victimization and Health Among Heterosexual and Lesbian Women

Research has shown that the prevalence of early, frequent, and more severe victimization is higher among lesbians than heterosexual women [17]. In a study comparing sexual minorities to their heterosexual siblings, Balsam and colleagues discovered that lesbians, compared to their heterosexual sisters, reported higher rates of adult sexual assault (16% vs. 8%), childhood physical abuse (16% vs. 11%), and childhood sexual abuse (44% vs. 30%) [18]. Similarly, Stoddard and colleagues examined rates of self-reported physical and sexual abuse among lesbians and their heterosexual biological sisters. They noted similar disparities in that more lesbians reported physical and sexual abuse when compared to their heterosexual sisters [19]. Given that siblings share a common familial environment, these studies suggest that children who later identify as a sexual minority may be specifically targeted for abuse within their own family unit. Other research shows that compared with their heterosexual peers, sexual minority women experience more severe and frequent childhood abuse, including sexual abuse starting at an earlier age and lasting for a longer duration [7], [20]. These disparities in victimization are of concern, yet few studies have examined the impact of victimization on physical health among sexual minority women.

The scarcity of research on differences in physical health between sexual minorities and heterosexuals is mainly due to the lack of data [21]. The limited evidence available suggests that sexual minority women, compared to heterosexual women, are at greater risk of developing some cancers and immune system deficiencies [22]. Asthma and obesity are other examples of disease conditions where physical health disparities are apparent among sexual minority populations [23], [24], [25]. Given that no substantiated biological differences exist between lesbian and heterosexual women, it may be that environmental exposures, such as victimization, help to explain health disparities. Individuals who experience more frequent and severe victimization are at greater risk of developing health problems over the lifespan than individuals who do not have these experiences [9]. For the present study, we examined if the impact of victimization on physical health differs between heterosexual and lesbian women.

### The Present Study

The aims of the present study are the following: 1) As an extension of the literature we hypothesize that childhood and adulthood victimization experiences (CSA, CPA, ASA, APA) will be positively associated with physical health problems among heterosexual and lesbian women. 2) We examine whether sexual identity moderates the relationship between victimization and health.

## Method

### Chicago Health and Life Experiences of Women (CHLEW)

In 2001–2002, the Chicago Health and Life Experiences of Women (CHLEW) study recruited 447 women from Chicago and surrounding suburban areas. In order to be eligible, participants were required to be English-speaking females, 18 or older, and to self-identify as lesbian. A unique aspect of this study was its recruitment strategies and methods that targeted women previously underrepresented in studies of lesbian health (e.g., older lesbians and lesbians with lower education and income levels). In addition, many earlier studies relied on recruitment from public social gatherings at bars or other large social events, resulting in samples that likely over-represented women who drink more heavily. The CHLEW study used a variety of recruitment methods, including advertisements targeted at public social network locations and events (e.g., churches and local community social clubs). The study was also advertised in newspapers and flyers that were posted in bookstores and passed from individual to individual within groups of friends and communities. Interested individuals were asked to call the project office for a screening interview to determine their eligibility.

Female interviewers met participants in private settings (typically the participant's home). Face-to-face interviews were approximately 90 minutes in length. Interviewers were extensively trained in both general and study-specific interviewing techniques, with particular attention to potentially sensitive topics such as child abuse and suicide. Before the interview began, participants were asked to read and sign a consent form, which described the purpose and procedures of the study. The consent form (of which a copy was given to each participant to take home) included a list of local and national resources with telephone numbers of domestic violence and child abuse hotlines, Alcoholics Anonymous, and other relevant mental health resources. Further, it was made clear to the participants that referrals for psychological help were available if necessary by the PIs on the study, including trained clinical psychologists and psychiatric nurses. Participants received $35 for taking the time to participate in the study. Greater detail about the study's design and methods can be found elsewhere [17].

### National Study of Health and Life Experiences of Women (NSHLEW)

The heterosexual comparison group consisted of a subsample of women from the 2001 survey of the National Study of Health and Life Experiences of Women (NSHLEW). The NSHLEW is a 20-year longitudinal study of women's drinking behaviors consisting of 5 waves of interview data between 1981and 2001 among nationally representative samples of adult U.S. women. Data for the current study are from a subsample of women interviewed in 2001(total N = 1,126). As with the CHLEW sample, face-to-face interviews were conducted by female interviewers in private settings. Interviewers were from the National Opinion Research Center and were extensively trained in general and study-specific interviewing techniques. The interviews were approximately 75minutes in length. More information about the NSHLEW design and methods can be found elsewhere [26], [27], [28].

### Ethics Statement

Study methods and procedures for the CHLEW and the NSHLEW were approved by the Institutional Review Boards of the University of Illinois at Chicago and the University of North Dakota, respectively.

### Analytic Samples of NSHLEW and CHLEW Women

In order to maximize comparability of the CHLEW and NSHLEW sample, participants from the two studies were selected based on several criteria. The first criterion was residence: since the CHLEW sample was recruited primarily from Chicago and surrounding suburbs, NSHLEW participants residing in large or medium-sized urban areas and surrounding suburbs were selected. Second, to increase comparability of age between the two samples, only women aged 21–70 were included in the pooled sample. Further, the same measures were used in both samples to facilitate comparability. After selection based on these criteria, the trimmed dataset included 405 women in the CHLEW sample and 548 women in the NSHLEW sample. We ran all the analyses again with the weighted data from Wilsnack et al., 2008, and the results remained the same. (For additional detail about the selection procedure, see [28]).

For the current analyses we selected cases from the combined CHLEW/NSHLEW sample based on sexual identity. Participants in both studies were asked, “Recognizing that sexuality is only part of your identity, how do you define your sexual identity?” The five response categories ranged from “only heterosexual” to “only homosexual/lesbian/gay.” In the CHLEW sample, 288 women (71.1%) self-identified as exclusively lesbian, 106 (26.2%) as mostly lesbian, and 11 (2.7%) as bisexual. Given the similarity of demographic and other characteristics between the categories “only lesbian” and “mostly lesbian,” we combined these groups. In the trimmed NSHLEW sample, 482 women (88.0%) self-identified as exclusively heterosexual, 42 women (7.7%) as mostly heterosexual, 11 (2.1%) as bisexual, 5 (0.9%) as mostly lesbian/gay, and 8 (1.5%) as only lesbian. Due to the fewer number of bisexual women from NSHLEW and CHLEW, the 11 bisexual women from each sample were merged to create a larger group of bisexual women (n = 22). Given their small numbers we excluded NSHLEW women who identified as lesbian. Thus, the final analytic sample included 940 women (482 exclusively heterosexual women, 42 mostly heterosexual women, 11 bisexual women from the NSHLEW and 394 lesbian-identified women, 11 bisexual women from the CHLEW).

### Measures

#### Child sexual abuse (CSA)

CSA was assessed by a series of questions about sexual activities before the age of 18. Experiences were classified as CSA according to criteria developed by Wyatt [29]. Specifically, CSA was defined as (a) any intrafamilial sexual activity before the age of 18 that was unwanted by the participant or that involved a family member who was 5 or more years older than the participant, or (b) any extrafamilial sexual activity that occurred before the age of 18 that was unwanted by the participant or that occurred before the age of 13 and involved another person who was 5 or more years older than the participant. CSA was coded as a dichotomous variable, with “1” indicating the presence of CSA and “0” indicating its absence. Additional information about the CSA measure can be found elsewhere [30], [31].

#### Child physical abuse (CPA)

CPA was assessed by a series of questions. First, participants were asked, “When you were growing up, were you physically hurt or injured by your parents or other family members?” (1 =  “Never,” 2 =  “Rarely,” 3 =  “Sometimes” 4 =  “Often,” 5 =  “Very Often”). If a participant reported being physically hurt or injured (i.e., those who gave any response other than ‘never’) they were asked a subsequent question: “Do you feel that you were physically abused by your parents or other family members when you were growing up?” We coded the answer to the second question dichotomously (1 =  “Yes,” 0 =  “No”) and used this as the measure of CPA for all analyses.

#### Cumulative childhood victimization

A childhood victimization score was created based on the summed score of CSA and CPA. A score of 0 indicated that the participant experienced no childhood victimization, a score of 1 indicated that the participant experienced either CSA or CPA, and a score of 2 indicated that the participant experienced both CSA and CPA.

#### Adult sexual assault (ASA)

Participants were asked the following questions regarding ASA: “Since you were 18 years old was there a time when you experienced any unwanted/forced sexual activity?” and “Has your partner ever forced you to have sex?” Participants who answered yes to *either* question were considered to have experienced ASA (1 =  “Yes,” 0 =  “No”).

#### Adult physical assault (APA)

Participants were asked the following questions about APA: “Has anyone - excluding your partner - attacked you without a weapon but with the intent to kill or seriously injure you?”; “Has anyone - excluding your partner - attacked you with a gun, knife, or some other weapon?”; “Has your most recent partner ever physically abused you in the last 12 months?”; and “Has your most recent partner threatened to kill you, with a weapon or in some other way?” Participants who answered yes to *any* of the questions were considered to have experienced APA (1 =  “Yes,” 0 =  “No”).

#### Cumulative adulthood victimization

An adulthood victimization score was created by taking the sum of ASA and APA. A score of 0 indicated that an individual had not experienced ASA or APA. A score of 1 indicated that the participant had experienced either ASA or APA, and a score of 2 indicated that the participant had experienced both ASA and APA.

#### Cumulative lifetime victimization

A cumulative victimization score was created by summing the four types of lifetime victimization experiences (CSA, CPA, ASA, APA). A score of 0 indicated that the participant reported none of the four victimization experiences; a score of 4 indicated that the participant reported all four types of victimization.

#### Physical health problems

In the present study, cardiovascular conditions (i.e., hypertension and heart disease), metabolic conditions (i.e., thyroid disorders and diabetes), and immune disease (i.e., cancer) were selected for examination given that these are serious health conditions and associated with victimization in prior studies [3], [4], [5], [9], [14]. Participants were asked “Have you experienced any thyroid problems, hypertension, diabetes, heart disease?” Answers were coded for each condition (0 = “No,” 1 = “Yes”). They were also asked “Have you been diagnosed with breast cancer in your lifetime?” (0 = “No,” 1 = “Yes”). Because overall prevalence of these disease conditions was low in this sample, we summed responses to questions about each of the health problems to create an index of the total number of health problems.

#### Covariates

We adjusted all analyses for age, body mass index (BMI) calculated from self-reported height and weight, race/ethnicity, education, income, depression (as measured by the National Institute of Mental Health Diagnostic Interview Schedule), current smoking (“are you a current smoker”), and heavy drinking (2 or more drinks/day on average for the past 30 days) given that these variables are known to have strong associations with health [32], [33], [34]. For the ethnicity variable, due to the fewer number of individuals who were Asian/Pacific Islander, Native American, and Other Racial group, we grouped them into one category. Because the majority of the sample was Caucasian/White, they were coded as the reference group.

### Data analysis

Given that the count of physical health problems was skewed (skewness  = 2.24, kurtosis  = 5.86) we fitted Poisson regression models. The physical health outcome variable was also tested for overdispersion to assess whether there was more variability in the data than predicted by the variance, which results in the underestimation of standard errors [35]. We followed Field's criteria, whereby overdispersion is present if the dispersion parameter (ratio of goodness-of-fit chi-square to degrees of freedom) reaches or approaches two [36]. There was no evidence of overdispersion for any of the Poisson regression models tested in this study. The Incidence Rate Ratio (IRR) is the relative change in the incidence rate for a one-unit change in the predictor variable. The goodness-of-fit chi-square statistic was not significant for any of the analyses using a Poisson regression, which suggested a good fit of the models to the data. We tested moderation by creating an interaction term and included that term in the relevant regression models to see if the interaction term was significant [37]. Due to the relatively large sample size and a relatively small number of missing data, missing data were handled by listwise deletion. The greatest number of missing data came from the most sensitive questions, but the proportion of missing data was still relatively small (CSA – 5.9% missing, CPA – 0.2% missing, ASA – 1.8% missing, APA – 1.7% missing). Only 0.2% of data was missing for the physical health problems outcome variable. All analyses were conducted using the statistical software SPSS, Version 20 (IBM Statistics).

## Results

### Demographics

As shown in Table 1, heterosexual and lesbian samples differed significantly on several demographic variables. There was a greater proportion of racial/ethnic minorities (p<.001) in the lesbian and bisexual samples compared to the mostly heterosexual and heterosexual samples. This is consistent with the CHLEW's special efforts to recruit lesbians of color. Lesbians, bisexuals, and mostly heterosexuals were more highly educated than heterosexuals (p<.001). Lesbians and mostly heterosexuals were also more likely to be depressed and to report heavy drinking than bisexuals and heterosexuals (p<.001 and p = .024, respectively). Although a one-way ANOVA indicated that the groups differed in age (p = .004), post-hoc analysis with a Bonferonni adjustment showed that the heterosexual and lesbian samples did not differ on age (which was understandable given that we selected the analytic sample using a restricted age range of 21–70 years). The group also differed significantly on BMI (p = .004. Lesbians, on average, had higher BMIs than heterosexuals and mostly heterosexuals.

**Table 1:** Demographic and Prevalence of Victimization Experiences Comparison between Heterosexual and Lesbian Women.

Demographic Variable	Heterosexuals (n = 482) n (%)	Lesbians (n = 394) n (%)	Bisexual (n = 22) n (%)	Mostly Heterosexual (n = 42) n (%)
Age (SD)	38.31(12.74)	38.40 (10.57)	30.05 (7.67)	34.93 (14.43)
BMI** (SD) (p = .004)	26.56 (6.70)	27.93 (7.44)	29.01 (7.33)	24.89 (5.27)
Race/Ethnicity*** (p<.001)				
White	344 (71.4)	180 (45.7)	12 (54.5)	35 (83.3)
Black	78 (15.8)	112 (28.4)	5 (22.7)	4 (9.5)
Asian/Pacific Islander	10 (2.1)	9 (2.3)	0 (0)	0 (0)
Hispanic	43 (8.9)	78 (19.8)	3 (13.6)	2 (4.8)
Native American	3 (0.6)	1 (0.3)	0 (0)	0 (0)
Other	6 (1.2)	14 (3.6)	2 (9.1)	1 (2.4)
Income (p = .052)				
<$10,000	39 (8.1)	47 (11.9)	2 (9.1)	5 (11.9)
$10,000–$29,999	98 (20.3)	90 (22.8)	4 (18.2)	10 (23.8)
$30,000–$39,999	67 (13.9)	59 (15.0)	9 (40.9)	6 (14.3)
$40,000–$59,999	101 (21.0)	64 (16.2)	5 (22.7)	7 (16.7)
>$60,000	163 (33.8)	134 (34.0)	2 (9.1)	14 (33.3)
Education*** (p<.001)				
High school or less	154 (32.0)	52 (13.2)	4 (18.2)	7 (16.7)
Some college	176 (36.5)	109 (2 7.7)	10 (45.5)	23 (54.8)
Bachelor's degree	99 (20.5)	107 (27.2)	6 (27.3)	8 (19.0)
Advanced degree	53 (11.0)	126 (32.0)	2 (9.1)	4 (9.5)
Smoking* (1 = yes/0 = no) (p = .024)	126 (26.2)	118 (29.9)	12 (54.5)	10 (23.8)
Depression*** (1 = yes/0 = no) (*p*<.001)	134 (27.8)	220 (56.0)	4 (18.2)	24 (57.1)
Heavy Drinking* (1 = yes/0 = no) (p = .024)	101 (21.0)	111 (28.2)	7 (31.8)	15 (35.7)
Trauma^1^				
CSA***	150 (31.2)	203 (59.2)	15 (71.4)	18 (43.9)
CPA***	45 (9.36)	85 (21.6)	9 (40.9)	8 (19.0)
ASA (p = .076)	100 (20.8)	106 (28.0)	7 (31.8)	9 (21.4)
APA***	41 (8.6)	122 (31.8)	6 (28.6)	2 (4.8)

*p<.05.

**p<.01.

***p<.001.

Note: The total n slightly differs based on non-responses. For demographic information based on weighted data, refer to Wilsnack et al., 2012, Alvy et al., 2012.

1 For prevalence rates based on weighted data, refer to Wilsnack et al., 2012, Alvy et al., 2012. CSA =  Child Sexual Abuse; CPA =  Child Physical Abuse; ASA =  Adult Sexual Assault; APA =  Adult Physical Assault.

### Physical Health Problems

In the total sample, 26.0% women reported one or more of the following health problems: thyroid problems, hypertension, diabetes, heart disease, and breast cancer. Comparisons of the specific health conditions are provided in Table 2. Bisexuals and mostly heterosexual groups reported fewer health problems than heterosexuals and lesbians most likely due to their smaller sample size. When comparing just the lesbian and heterosexual groups, no disparities in health problems were found except for thyroid problems, where heterosexual women were more likely to report. The prevalence of health problems was similar for heterosexual (27.4%) and lesbian (25.7%) women. Due the much fewer number of health problems among bisexuals and mostly heterosexuals, and given their smaller sample size, we omitted bisexuals and mostly heterosexuals from the subsequent analysis examining the impact of victimization on health. Thus, the subsequent analyses only include lesbian and heterosexual women.

**Table 2:** Physical Health Problems Experienced by Heterosexual and Sexual Minority Women.

Health Variable	Heterosexuals (n = 482) n (%)	Lesbians (n = 394) n (%)	Bisexual (n = 22) n (%)	Mostly Heterosexual (n = 42) n (%)	*p*
Thyroid problems	55 (11.4)	27 (6.9)	1 (4.5)	7 (16.7)	.040
Hypertension	68 (14.1)	67 (17.0)	0 (0)	2 (4.8)	.030
Diabetes	24 (5.0)	18 (4.6)	1 (4.5)	0 (0)	.533
Heart disease	16 (3.3)	14 (3.6)	0 (0)	0 (0)	.512
Breast cancer	9 (1.9)	10 (2.5)	0 (0)	0 (0)	.591
Any health problem	132 (27.4)	101 (25.7)	2 (9.1)	9 (21.4)	.684
1 health problem	102 (21.2)	72 (18.3)	2 (9.1)	9 (21.4)	
2 health problems	23 (4.8)	24 (6.1)	0 (0)	0 (0)	
3+ health problems	7 (1.4)	5 (1.3)	0 (0)	0 (0)	

Note: The total n slightly differs based on non-responses. Prevalence rates are based on unweighted data, so these rates may not be generalizable to the national sample. Overall distribution of health problems were not significantly different across sexual identity (p = .12).

### Victimization

Table 1 shows the number and percent of women reporting each type of victimization. In general sexual minority groups (lesbian, bisexual, mostly heterosexual) were more likely to experience each type of victimization (p's<.05 for CSA, CPA, APA, p<.1 for ASA). When we compared only the lesbian and the heterosexual sample, significantly more lesbian than heterosexual women reported experiencing each type of victimization (p's<.05). Previously published data on this dataset shows that lesbian women had earlier age of onset of abuse, higher frequency and more severe abuse [17].

### Control Variables

It should be noted that of all the control variables, only age and BMI were consistent significant predictors of negative health outcomes (*p*<.001). Smoking, heavy drinking, depression, education, income and sexual identity were not significantly associated with health problems. Compared to White/Caucasians, Blacks were significantly more likely to report health problems (*p* = .03). However, this difference was not found for Asians, Hispanics, and Other Racial minority groups. See Model 1 in Table 3 for more details.

**Table 3:** Childhood and Lifetime Victimization as a Predictor of Health Problems: Multivariate Poisson regression models.

	Model 1		Model 2∧		Model 3∧		Model 4∧	
	(n = 850)		(n = 798)		(n = 829)		(n = 782)	
	*IRR_adj_* (*p*)	*95% CI*	*IRR_adj_* (*p*)	*95% CI*	*IRR_adj_* (*p*)	*95% CI*	*IRR_adj_* (*p*)	*95% CI*
**Demographics**								
Age	1.05(<.001)	1.04, 1.06						
BMI	1.06(<.001)	1.05, 1.08						
Depression	1.13(.296)	0.90, 1.43						
Heavy Drinking	1.11(.522)	0.82, 1.50						
Smoking	0.97 (.85)	0.73, 1.30						
Race (Black)^a^	1.35 (.03)	1.03, 1.78						
Race (Hispanic)^a^	0.96(.870)	0.60, 1.55						
Race (Other)^a^	0.40(.275)	0.80, 2.06						
Income	0.97 .523)	0.88, 1.07						
Education	1.01(.867)	0.88, 1.16						
Sexual Identity^b^	1.02 (.743)	0.90, 1.17						
**Childhood Victimization** c								
1 trauma type			0.91(.524)	0.69, 1.21				
2 trauma types			1.44(.056)	0.99, 2.10				
**Adulthood Victimization** c								
1 trauma type					0.98(.878)	0.76, 1.27		
2 trauma types					1.40(.107)	0.93, 2.07		
**Lifetime Victimization** c								
1 trauma type							0.93(.605)	0.69, 1.24
2 trauma types							1.10(.583)	0.78, 1.54
3 trauma types							1.08(.731)	0.69, 1.72
4 trauma types							2.38(<.001)	1.44, 3.95

a In comparison to Caucasian/White;

b 0 =  Heterosexual; 1 =  Lesbians;

c Trauma  = 0 as the reference group;

∧ Model controls for age, BMI, depression, heavy drinking, education, race, income, sexual identity.

### Victimization and Health – Independent Associations

Using separate Poisson regression models adjusting for demographic characteristics and health correlates, CSA, CPA, ASA and APA were not independently associated with physical health problems (data not shown).

### Cumulative Victimization and Health

#### Childhood victimization vs. adulthood victimization

Poisson regression modeling was used to examine the association between childhood victimization (i.e., the number of childhood trauma types [one, both, or neither CSA or CPA]) and health problems (Table 3, model 2). Results indicate that experiencing both CSA and CPA was a significant predictor of health problems (χ^2^ (*df* = 2)  = 7.83, p = .02). Participants who experienced *both* CSA and CPA were 1.44 (95% CI [0.99, 2.10]) times as likely to report health problems than those who reported no childhood victimization. The same analysis was conducted for adult victimization, (i.e., the number of adulthood trauma types [one, both, or neither ASA or APA]) and health problems but no statistically significant relationships were found.

#### Cumulative lifetime victimization

We examined the relationship between health problems and the total number of types of lifetime victimization experienced (i.e., CSA, CPA, ASA, APA), using a Poisson regression similar to the above model. Cumulative lifetime victimization was significantly associated with physical health problems (χ^2^ (*df* = 4)  = 14.83, p = .005). Participants who experienced all four types of victimization were 2.38 (95% CI [1.44, 3.95]) times as likely to report health problems as those who reported no victimization in either childhood or adulthood.

### Sexual Identity and Health

We examined the data to determine whether the relationships between individual types of lifetime victimization and physical health differed based on sexual identity. We conducted separate multiple regression analyses in which each type of victimization (CSA, CPA, ASA, APA) and cumulative victimization (childhood, adulthood, and lifetime) was associated with having physical health problems while including an interaction term for sexual identity x victimization experience. None of the interaction terms were significant, indicating that victimization experiences conferred increased physical health risks regardless of sexual identity.

## Discussion

In support of our hypothesis, lifetime victimization was associated with physical health problems, consistent with previous research. Specifically, we found that women who experienced two types of childhood victimization (CSA and CPA) had a 44% increased risk of health problems compared to those who had not experienced childhood victimization. We also found evidence for the cumulative impact of victimization. Women who experienced all four types of lifetime victimization (CSA, CPA, ASA, APA) showed a nearly 240% elevated risk for health problems compared to peers who had not experienced victimization in their lifetime. These findings suggest that adult victimization experiences contribute to the risk of health problems in the presence of prior adversity and may exacerbate disease processes that have already been started. These findings are consistent with those of Fagundes and colleagues [3] on the cumulative effects of adversity impacting the course of disease. Findings from this study underscore the importance of examining childhood and adult victimization experiences separately, as well as in combination.

It is important to note that our findings did not replicate previous findings [4], [5] on the impact of a single type of victimization experience on physical health for CSA, CPA, ASA or APA. A possible reason that we did not replicate prior findings may be that our victimization measures were overly broad. It may be that specific characteristics of each victimization experience (e.g., severity or frequency) moderate the impact of victimization on physical health. For instance, it is possible that the impact of CSA on physical health may be moderated by the frequency of the CSA experience, such that those who have experienced a greater frequency of CSA will have greater health risks. While these hypotheses were beyond the scope of this paper, this is an interesting avenue for future researchers to explore.

### Interactive Effects of Sexual Identity and Victimization on Health

Prior literature reports disparities in mental health and in some physical health conditions between sexual minorities and heterosexuals [6], [22]. In this study, all sexual minority women (i.e., lesbian, bisexual and mostly heterosexual) reported higher rates of all forms of victimization (CSA, CPA, ASA, APA). However, we did not find sexual identity-related disparities in physical health, which was surprising. The reason for this latter finding should not be an issue of low power, given that our sample is sufficiently large enough to detect meaningful effects in a Poisson Regression (N = 850 to 782) [38]. We also examined possible interaction effects between sexual identity and four types of victimization experiences on physical health. Despite not finding health disparities between heterosexual and lesbian women in our sample, it is still possible to test for moderation. Moderation can exist in the absence of main effects. Sexual identity did not moderate the relationship between trauma and physical health. Our results suggest that lifetime victimization confers significant health risks regardless of sexual identity. It may be the case that we did not find moderation because the health variable may not have been sensitive enough to detect differences between lesbians and heterosexual women. However, our findings are consistent with recent research by Balsam and colleagues who examined the impact of sexual revictimization on mental health among lesbians, gay men, and heterosexual women [39]. The authors found that CSA was associated with elevated rates of adult rape for all three groups, and that revictimization showed comparable associations with mental health for all three groups. This study provides further support for the authors' findings that the relationship between victimization and health appears comparable across sexual identity. It may also be possible that additional protective factors, such as increased social support among sexual minority groups, may alleviate some of the negative impact of victimization on health [40], [41].

A prominent model of minority stress [42] suggests that sexual minority individuals may be at higher risk for poor health outcomes, given elevated levels of chronic stress resulting from the social stigma associated with being a member of this minority group. An inherent assumption in this model is that it is not sexual identity *per se* or some associated biological or physiological difference that causes susceptibility to negative outcomes, but rather, adverse social factors that collectively confer risk upon a sexual minority person. For instance, evidence shows that sexual minority individuals are often targeted for victimization from peers, and sometimes even family members [6], [20]. Roberts and colleagues found that sexual minorities were at significantly elevated risk for posttraumatic stress disorder compared to heterosexuals [7]. Their results suggest that elevated exposure to traumatic events and violence, beginning from an earlier age than their heterosexual peers, rather than sexual identity, account for this disparity. Studies have established a dose-response relationship between victimization and health later in life [9]. Using data from the National Epidemiologic Study of Alcohol and Related Conditions, Hughes and colleagues found that substance abuse was higher among LGBT individuals, but victimization (particularly 2 or more experiences) explained the higher prevalence of substance abuse regardless of sexual identity [43]. Our findings contribute to the growing body of evidence suggesting that it is not inherent characteristics of minority status that contribute to poor health, but rather social factors (e.g., being a target of abuse) that confer health risk [7]. The good news is that social factors can be addressed by research, societal and policy change related to reducing disparities in violence and victimization aimed at sexual minority individuals.

### Limitations

It is important to note several limitations of this study. First, our measure of health problems was a sum of 5 dichotomous variables assessing presence/absence of disease. It is possible that trauma more strongly influences the severity or timing of health problems rather than simply the presence of health problems. For instance, two people may both be diagnosed with diabetes but its severity could differ greatly from one person to the next [44]. Based on prior research, we know that the impact of victimization on physical health may appear more readily in the presence of concurrent life stress [3], [9]. Understanding how trauma impacts the severity and onset of disease carries important policy implications for the type and timings of intervention research. Second, physical health problems included in these analyses were self-reported. It is possible that we did not find high rates of health problems due to a self-report bias. However, the low rates of health problems also may reflect the nature of our sample – a non-clinical and relatively young (mean age  = 38 years) group – which would be expected to be in reasonably good health. The authors of several meta-analytic reviews report that much of the literature on victimization and health is based on self-reported information and that rates of health problems are higher among clinical samples [4], [5]. Longitudinal studies that include objective measures of health are necessary to observe this process as it unfolds. Third, victimization experiences were also assessed as dichotomous variables (i.e., presence/absence). Other components of victimization experiences, such as its frequency, duration, and severity have also been shown to have an impact on health [4], [5]. The additive model in this study does not fully capture the complexities of the relationships between characteristics of victimization (e.g., perpetrator status, age of onset, frequency and duration) and health outcomes. Victimization experiences were also self-reported; experiences that occurred earlier in life, in particular, may be prone to recall bias. The possibility of underreporting should always be considered when interviews include sensitive topics such as sexual and physical victimization.

Another limitation involves sample characteristics. The heterosexual women were from a national probability sample and the lesbian women were from a Chicago-area community-based sample. We attempted to maximize the comparability of the two samples by using the same age range, limiting the heterosexual sample to women living in or near large cities, and controlling for major demographic variables. The use of identical interview questionnaires, measures, and interview procedures also helps to mitigate differences between samples. Furthermore, due to subsample size, we omitted bisexual and ‘mostly heterosexual’ women from the regression analyses, given insufficient power to detect meaningful effects. However, it is clear from the high percentage of bisexual and mostly heterosexual women reporting all trauma types, that these subgroups are important samples that should be targeted for future research due to their unique experiences. For instance, bisexual individuals are not only stigmatized by the heterosexual community, but also from the gay and lesbian communities, which often results in worse physical and mental health for these groups [45].

Because the study used cross-sectional data it is not possible to determine causality. Childhood events by definition occurred prior to age 18, and five of the six health problems were assessed using a ‘past five years' time frame (the question about breast cancer asked about lifetime diagnosis), making it reasonable to assume that childhood events preceded the occurrence of health problems; however, adult victimization may have occurred after the health problems began. Additionally, due to the lack of longitudinal data, it is difficult to establish which variables cause the disparities in victimization experiences. Our focus in this paper was to examine the cumulative impact of victimization across childhood, independently from adulthood, and in combination. Despite the limitations, we believe that this paper conveys important information about the additive effects of childhood and adulthood victimization among both heterosexual and lesbian women.

### Implications for Treatment and Prevention

Future studies are encouraged to examine the health impact of multiple victimization experiences in combination with other stressful experiences that sexual minority individuals face, such as sexual identity-based discrimination and harassment. In addition to better understanding the similarities and differences in the health trajectories of heterosexual and lesbian women who experience victimization, research is urgently needed to understand why sexual minority women appear to be at heightened risk for victimization, and to then use these findings to develop prevention and early intervention strategies. Further, it is important to extend this area of research to include sexual minority males. Much of the research on victimization uses data gathered from self-reports of survivors. Yet to move toward the goal of developing effective intervention programs, understanding why perpetrators target sexual minority persons must also be investigated and addressed. Finally, researchers should be encouraged to explore protective factors that may mitigate some of the health risks associated with victimization, such as social support, therapy and access to comprehensive culturally competent health care providers.
